# Extracellular Vesicles from NMN Preconditioned Mesenchymal Stem Cells Ameliorated Myocardial Infarction via miR-210-3p Promoted Angiogenesis

**DOI:** 10.1007/s12015-022-10499-6

**Published:** 2023-01-25

**Authors:** Yanan Pu, Chunyu Li, Xin Qi, Rui Xu, Liyang Dong, Yi Jiang, Qingyun Gong, Di Wang, Rong Cheng, Cheng Zhang, Yan Chen

**Affiliations:** 1 Outpatient & Emergency Management Department, The First Affiliated Hospital of Nanjing Medical University, Nanjing, 210029 Jiangsu China; 2 Women & Children Intensive Care Unit, The First Affiliated Hospital of Nanjing Medical University, Nanjing, 210029 Jiangsu China; 3 State Key Lab of Reproductive Medicine, Jiangsu Key Laboratory of Pathogen Biology, Department of Pathogen Biology and Immunology, Center for Global Health, Nanjing Medical University, Nanjing, 211166 Jiangsu China; 4 Department of Nuclear Medicine, The Affiliated Hospital of Jiangsu University, Zhenjiang, 212000 Jiangsu China; 5 Women & Children Central Laboratory, The First Affiliated Hospital of Nanjing Medical University, Nanjing, 210029 Jiangsu China; 6 Emergency Management Department, School of Health Policy & Management, Nanjing Medical University, Nanjing, 211166 Jiangsu China; 7 Research Institute of Health Jiangsu, Nanjing Medical University, Nanjing, 211166 Jiangsu China

**Keywords:** NMN, Mesenchymal stem cells, EVs, Angiogenesis, miR-210-3p

## Abstract

Mesenchymal stem cell-derived extracellular vesicles (MSCs-EVs) possess cardioprotection in acute myocardial infarction. Nevertheless, the therapeutic intervention potential and the molecular mechanism of EVs from NMN (Nicotinamide mononucleotide) preconditioned hUCMSCs (N-EVs) in acute myocardial infarction remains unknown. In the present study, EVs from hUCMSCs (M-EVs) and N-EVs were identified by electron microscopy, immunoblotting and nanoparticle tracking analysis. Compared with M-EVs, N-EVs significantly increased the proliferation, migration, and angiogenesis of HUVECs. Meanwhile, N-EVs markedly reduced apoptosis and cardiac fibrosis and promoted angiogenesis in the peri-infarct region in the MI rats. A high-throughput miRNA sequencing and qPCR methods analysis revealed that miR-210-3p was abundant in N-EVs and the expression of miR-210-3p was obviously upregulated in HUVECs after N-EVs treated. Overexpression of miR-210-3p in HUVECs significantly enhanced the tube formation, migration and proliferative capacities of HUVECs. However, downregulation of miR-210-3p in HUVECs markedly decreased the tube formation, migration and proliferative capacities of HUVECs. Furthermore, bioinformatics analysis and luciferase assays revealed that EphrinA3 (EFNA3) was a direct target of miR-210-3p. Knockdown of miR-210-3p in N-EVs significantly impaired its ability to protect the heart after myocardial infarction. Altogether, these results indicated that N-EVs promoted the infarct healing through improvement of angiogenesis by miR-210-3p via targeting the EFNA3.

**Graphical Abstract:**

Created with Biorender.com.

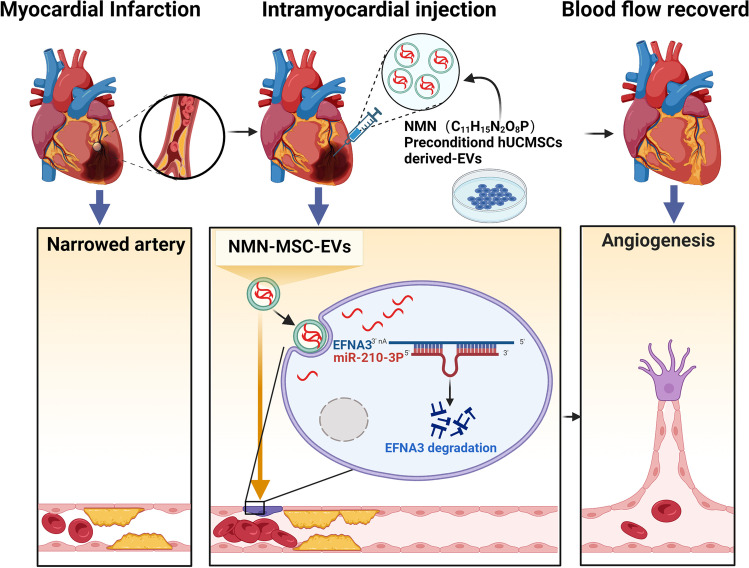

**Supplementary Information:**

The online version contains supplementary material available at 10.1007/s12015-022-10499-6.

## Introduction

Acute myocardial infarction (AMI), accounts for 80% of death in patients with ischemic heart disease worldwide, which is the leading cause of mortality around the world in 2016 and its prevalence continues to increase every year[1, 2]. The primary pathological cause of AMI are myocardial ischemia and hypoxia following coronary artery occlusion. Previous studies have revealed that the upregulation of angiogenesis attenuated ventricular remodelling and improved cardiac function after myocardial infarction[3–5]. Therefore, therapeutic angiogenesis is a promising approach in ischemic myocardium.

MSCs are stromal stem cells from adult tissues and the research about MSCs from bone marrow, adipose tissue, the umbilical cord is the most common. MSCs have a wide and good therapeutic effect and has been applied to the treatment of many diseases, such as neurological disease[6, 7], respiratory disease[8, 9], fracture healing[10–12] and cardiovascular disease[13, 14], etc. Recent studies indicated that MSCs achieved their cardioprotective effect mainly through secreting paracrine factors including Extracellular vesicles (EVs) [15–17]. EVs are small membrane vesicles, originating intracellularly from a wide array of cell types, which contribute to donor cell-mediated biological effects by transferring a subset of proteins, lipids, and nucleic acids[18]. Compared with MSC, EVs not only retain the same curative effect as the parent cell, but also have the advantages of easy storage and transportation, easy internalization, low immunogenicity, and low tumorigenesis risk [19]. A growing number of research studies have shown transplantation of MSC or MSC-EVs exhibited similar therapeutic effects through protected against cell apoptosis and enhanced blood flow recovery in AMI [20, 21]. Thus, EVs therapy is a promising cell-free therapy for ischemic cardiomyopathy.

When cells are stimulated with a variety of environmental stimuli, the content of EVs will also change and this would influence their biological effects. Therefore, it is important to optimize MSC-EVs in vitro to improve their therapeutic efficacies. Various strategies have been adopted to enhance their cardioprotective effects, including genetic modification, hypoxia and drug pretreatment [22–24]. Nicotinamide mononucleotide (NMN) is a critical precursor of nicotinamide adenine dinucleotide (NAD^+^), which is essential for regulating multiple physiological and biochemical processes. Previous studies have observed that administering NMN in vivo could efficiently protect the heart against ageing-induced, doxorubicin treatment and post-ischemia-induced damage. Therefore, we hypothesized that the anti-AMI effect of MSC-EVs might be enhanced by NMN pretreatment.

In this study, our results showed that N-EVs significantly increased the proliferation, migration, and angiogenesis of HUVECs than M-EVs in vitro. In vivo rat experiments showed that N-EVs prominently reduced ventricular remodelling and improved cardiac function after myocardial infarction. Meanwhile, N-EVs significantly reduced apoptosis and cardiac fibrosis and promoted angiogenesis in the peri-infarct region. MiRNA sequencing and qPCR methods analysis revealed that miR-210-3p was abundant in N-EVs (vs. M-EVs). However, inhibition of miR-210-3p abolished the cardioprotection conferred by N-EVs. These results suggested that miR-210-3p-mediated N-EVs has a potential therapeutic effect on ischemic heart.

## Results

### Characterization of M-EVs and N-EVs

**Fig. 1:**
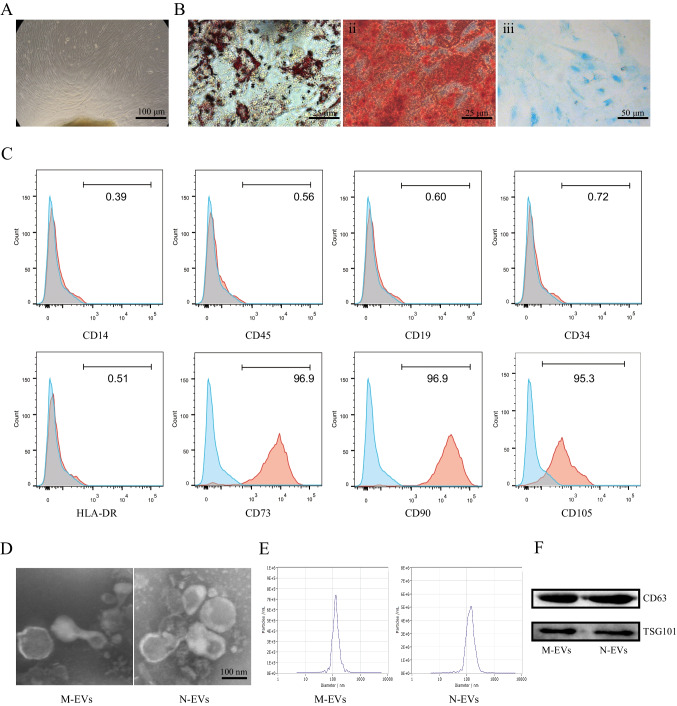
Isolation and characterization of hUCMSCs, M-EVs and N-EVs. **A** Morphology of hUCMSCs was visualized under light microscopy. **B** Representative photographs of osteocyte (i), adipocyte (ii) and chondrocyte (iii) differentiation of hUCMSCs cultured in the differentiation media. **C** Surface markers related to hUCMSCs by flow cytometry analysis. HUCMSCs were CD73^+^, CD90^+^, CD105^+^, CD19^−^, CD34^−^, CD45^−^, CD14^−^ and HLA-DR^−^ by flow cytometry. **D** Typical images of TEM of M-EVs and N-EVs. **E** The particle size distribution and concentration of M-EVs and N-EVs by NTA. **F** Western blot analysis of TSG101 and CD63 expression in M-EVs and N-EVs

The hUCMSCs were confirmed on the basis of the criteria defined by International Society for Cellular Therapy [25]. First, we have performed CCK8 assays to assess the proliferation of hUCMSCs and the results of CCK8 assays indicated that hUCMSCs has a promising clonogenic potential with time-dependence (Fig. S1). The hUCMSCs were cultured in osteogenic, chondrogenic, or adipogenic media and then were stained with Alizarin Red, Oil Red O and Alcian Blue, respectively. These results confirmed that hUCMSCs have the properties with multi-lineage differentiation potential to differentiate into osteocytes, adipocyte and chondrocyte (Fig. 1A, B). These cells also present CD73, CD90, and CD105 surface antigens and lack expression of CD45, CD14, CD34, CD19, HLA-DR (Fig. 1C). The hUCMSCs were cultured in culture medium with or without NMN for 48 h and then we also evaluated the effect of NMN on differentiation potential and phenotype of hUCMSCs. Our experimental results confirmed that hUCMSCs remained the properties with multi-lineage differentiation potential to differentiate into osteocytes, adipocyte and chondrocyte after NMN pretreatment (Fig. S2A). Flow cytometric analysis showed that these cells also present CD73, CD90, and CD105 surface antigens and lack expression of CD45, CD14, CD34, CD19, HLA-DR after NMN pretreatment (Fig. S2B). Together, these results demonstrated that the treatment with NMN couldn’t modified the hUCMSCs phenotype and differentiation capacities. After NMN pretreatment, the hUCMSCs were cultured in serum free culture medium supplemented with 1% of PS for an additional 24 h. Subsequently, the supernatant was collected for EVs isolation. M-EVs and N-EVs were identified and characterized by using transmission electron microscope (TEM), nanoparticle tracking analysis (NTA) and western blot. TEM images showed M-EVs and N-EVs were cup- or disc-shaped. No morphological difference between M-EVs and N-EVs was observed with regard to their shape (Fig. 1D). NTA analysis revealed that the average diameter of M-EVs and N-EVs about 140 nm (Fig. 1E). Western blot analysis revealed that several EVs markers including tumor susceptibility gene 101 (TSG101) and Cluster of differentiation 63 (CD63) [17] were detected in these EVs (Fig. 1F). The above results suggested that hUCMSCs and EVs meets the specified criterion.

### N-EVs Significantly Enhanced Angiogenesis in HUVECs more than M-EVs

**Fig. 2:**
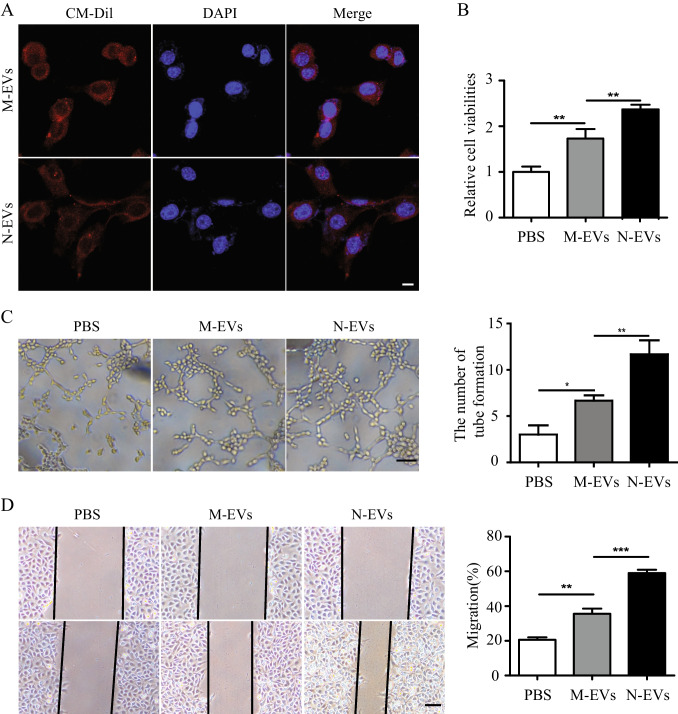
N-EVs significantly enhanced angiogenesis in HUVECs more than M-EVs. **A** Confocal images showed that red fluorescence of dye Dil labeled EVs were endocytosed by HUVECs. scale bar = 25 μm. **B** Cell proliferation of HUVECs incubated with PBS, M-EVs, N-EVs by CCK-8. **C** Tube formation of HUVECs incubated with PBS, M-EVs, N-EVs and quantification analysis. scale bar = 25 μm. **D** Migration was monitored for 12 h after scratching in HUVECs cultured with PBS, M-EVs, N-EVs and quantification analysis. scale bar = 25 μm. Data were expressed as the mean ± SD, ^*^P < 0.05, ^**^P < 0.01, ^***^P < 0.001

In order to evaluate the effect of M-EVs and N-EVs on HUVECs, we first confirmed that HUVECs could take up M-EVs and N-EVs by co-culturing CM-Dil dye-labeled EVs with HUVECs (Fig. 2A). Subsequently, we performed cell proliferation assay, cell migration assay, tube formation assays in HUVECs to assess the effect of M-EVs and N-EVs on angiogenesis in vitro. CCK-8 experiment results showed N-EVs markedly promoted the proliferation of HUVECs more than M-EVs (Fig. 2B). The tubule formation assays results revealed that N-EVs significantly increased the number of loops formed by the tubules compared with M-EVs group (Fig. 2C). Consistently, the migration distance of the HUVECs treated with N-EVs was significantly greater compared to that of the HUVECs treated with M-EVs (Fig. 2D). In summary, these results indicated that N-EVs has a better effect enhanced angiogenesis in HUVECs than M-EVs in vitro.

### N-EVs Effectively Protected Cardiac Function in a Rat Model of MI more than M-EVs

**Fig. 3:**
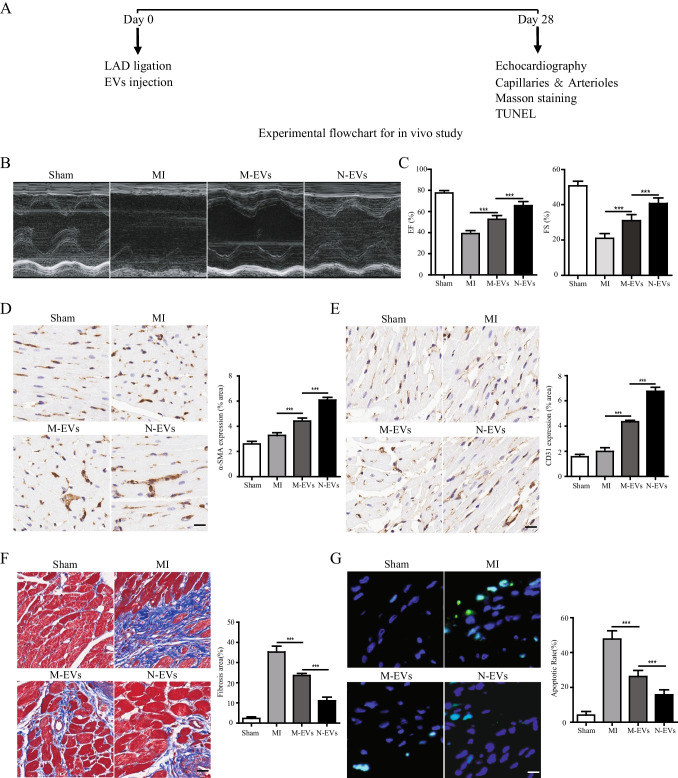
N-EVs effectively protect cardiac function in a rat model of MI more than M-EVs. **A** The flowchart of experimental design in vivo. **B** Representative echocardiogram of rat heart in different groups at 28 days post-MI. scale bar = 25 μm. **C** Significantly enhanced LVEF, LVFS in rats transplanted with N-EVs compared with other groups. **D** α-SMA positively stained arterioles in the infarct area week 4 after MI. scale bar = 25 μm. **E** CD31 positively stained capillaries at the border zone 4 weeks post MI. scale bar = 25 μm. **F** Representative transverse heart sections analysed with Masson Trichrome staining at 4 weeks after MI. scale bar = 25 μm. **G** TUNEL staining at the border zone 4 weeks after MI. scale bar = 25 μm. n = 5 for each group. Data were expressed as the mean ± SD. ^*^P < 0.05, ^**^P < 0.01, ^***^P < 0.001

In order to evaluate the protective effect of N-EVs cardiac function in vivo, PBS, M-EVs, N-EVs were injected at the border of infarction area in rats (Fig. 3A). Four weeks after MI, echocardiography showed that LVEF and LVFS significantly increased in the N-EVs group compared with PBS and M-EVs groups (Fig. 3B, C). The role of N-EVs was also investigated to determine any therapeutic potential on angiogenesis, fibrosis, and anti-apoptosis in vivo. In order to explore the mechanism underlying the enhanced cardiac function conferred by EVs therapy in rat. We used antibodies against α-SMA and CD31 for immunohistochemistry to show arterioles and capillaries. The density of small arteries in the N-EVs group was significantly increased compared with the M-EVs and AMI groups 4 weeks after infarction, and the capillary density also had a similar trend (Fig. 3D, E). Angiogenesis also contribute to reduce fibrosis size and cell apoptosis in infarcted hearts. Masson staining results indicated that the area of fibrosis clearly decreased in the N-EVs group compared with PBS, M-EVs groups (Fig. 3F). TUNEL showed that apoptotic cells significantly decreased in the N-EVs group compared with PBS, M-EVs groups (Fig. 3G). Thus, these results indicated that N-EVs could promoted angiogenesis, which may be one of the mechanisms to enhance the ability of the heart to repair.

### MiR-210-3p Expression Significantly Increased in N-EVs Compared to M-EVs

**Fig. 4:**
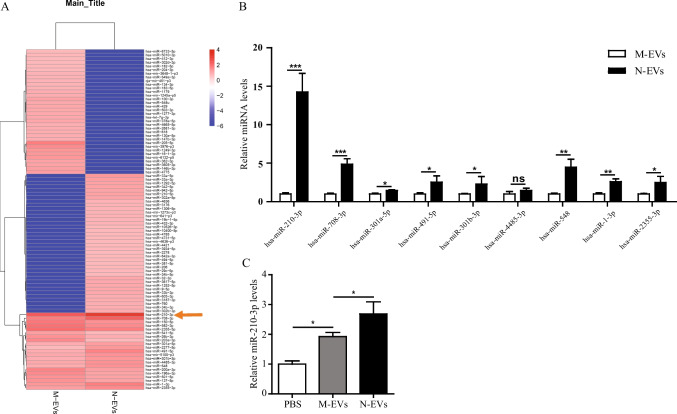
MiR-210-3p was enriched in N-EVs and delivered to HUVECs. **A** Heat map of the top nine most abundant miRNAs in M-EVs and N-EVs by miRNA-seq. **B** QRT-PCR analysis of nine most abundant miRNAs in M-EVs and N-EVs. **C** QRT-PCR analysis of miR-210-3p in HUVECs. Data were expressed as the mean ± SD, ^*^P < 0.05, ^**^P < 0.01, ^***^P < 0.001

To explore the molecular mechanisms by which N-EVs can protect heart in AMI rats, miRNAs sequencing was performed on M-EVs and N-EVs. It was notable that there were nine abundant miRNAs, including hsa-miR-210-3p, hsa-miR-708-3p, hsa-miR-301a-5p, hsa-miR-491-5p, hsa-miR-301b-3p, hsa-miR-4485-3p, hsa-miR-548, hsa-miR-1-3p and hsa-miR-2355-3p in N-EVs versus M-EVs (Fig. 4A). Among them, the expression of miR-210-3p was the highest in N-EVs than M-EVs determined by qRT-PCR (Fig. 4B). To confirm miR-210-3p could be delivered to HUVECs by N-EVs treatment, qRT-PCR tested the expression of miR-210-3p was clearly up-regulated in HUVECs after N-EVs treatment (Fig. 4C). Taken together, these results suggested that miR-210-3p expression is significantly elevated in N-EVs compared to M-EVs and could be efficiently delivered to HUVECs.

### Function of miR-210-3p on Angiogenesis of HUVECs in vitro

**Fig. 5:**
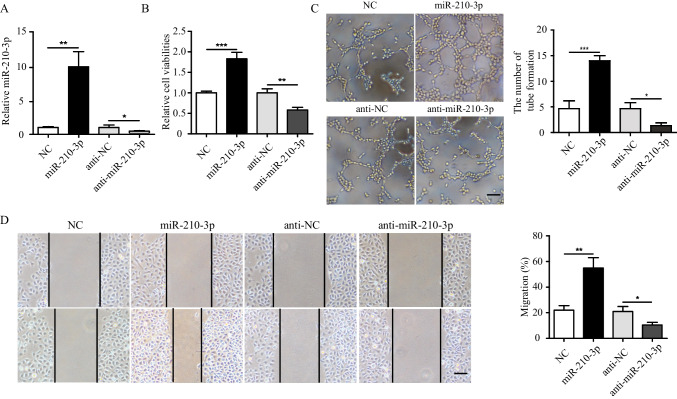
Function of miR-210-3p on angiogenesis of HUVECs in vitro. **A** MiR-210-3p expression was detected in HUVECs were transfected with miR-210-3p mimics/inhibitor or controls (NC) by qRT-PCR. **B** Cell proliferation of HUVECs were transfected with miR-210-3p mimics/inhibitor or controls (NC). **C** Tubule formation of HUVECs were transfected with miR-210-3p mimics/inhibitor or control (NC) and quantification analysis. scale bar = 25 μm. **D** Migration was monitored for 12 h after scratching in HUVECs were transfected with miR-210-3p mimics/inhibitor or control (NC) and quantification analysis. scale bar = 25 μm. Data were expressed as the mean ± SD, ^*^P < 0.05, ^**^P < 0.01, ^***^P < 0.001

Gain- and loss- of function studies were carried out to identify the effects of miR-210-3p on angiogenesis in HUVECs in vitro. HUVECs were transfected with miR-210-3p mimics, miR-210-3p inhibitor, and their negative controls (NC) successfully and were validated by qRT-PCR (Fig. 5A). We found that HUVECs were transfected with miR-210-3p mimics (miR-210-3p) markedly promoted the proliferation of HUVECs. However, when miR-210-3p was inhibited in HUVECs, the proliferation of HUVECs significantly reduced (Fig. 5B). Similarly, the overexpression of miR-210-3p in HUVECs significantly increased the number of loops formed by the tubules and the migration distance of HUVECs. In contrast, the number of loops formed by the tubules and the migration distance of HUVECs treated with miR-210-3p inhibitor obviously decreased (Fig. 5C, D). Overall, these results indicated that miR-210-3p plays a key role on angiogenesis of HUVECs.

### MiR-210-3p Enhanced Angiogenesis of HUVECs via EFNA3

**Fig. 6:**
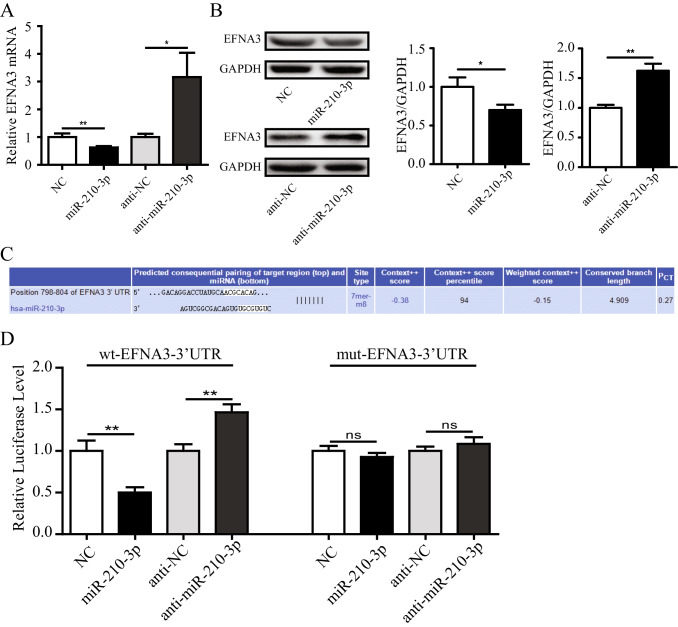
MiR-210-3p enhanced angiogenesis of HUVECs via EFNA3. **A** The mRNA levels of EFNA3 in HUVECs were transfected with miR-210-3p mimics/inhibitor or their controls. **B** The protein levels of EFNA3 in HUVECs were transfected with miR-210-3p mimics/inhibitor or their controls. **C** EFNA3 and miR-210-3p-binding site map. **D** MiR-210-3p dramatically suppressed the luciferase activity that carried wild-type but not mutant 3’-UTR of EFNA3 in HUVECs. Data were expressed as the mean ± SD. ^*^P < 0.05, ^**^P < 0.01

To further identify the potential signal pathway involved with miR-210-3p biological activity, according to the previous literatures [26, 27] and TargetScanHuman, an online prediction of microRNA targets (http://www.targetscan.org/), EFNA3 might be a target of miR-210-3p in HUVECs. To confirm this hypothesis, qRT-PCR was used to tested the expression of EFNA3 mRNA in HUVECs, which were transfected with miR-210-3p mimics/inhibitor and their NC. As shown in Fig. 6A, our results showed that overexpression of miR-210-3p could significantly down-regulated EFNA3 mRNA in HUVECs. However, consistent with expectations, inhibition of miR-210-3p could significantly up-regulated EFNA3 mRNA in HUVECs. Consistent with this trend were EFNA3 protein expression in HUVECs (Fig. 6B). To further identify whether miR-210-3p directly binded the 3’-UTR region of EFNA3, we conducted chimeric constructs, which harbor luciferase mutant 3’-UTR sequence (mt-EFNA3-3’-UTR) or wild-type 3’-UTR sequence (wt-EFNA3-3’-UTR). Overexpression of miR-210-3p repressed the luciferase activity of the reporter gene within the wild-type construct but not the mutant EFNA3-3’-UTR construct, while miR-210-3p inhibition increased the luciferase activity (Fig. 6C, D). Together, these results suggested that miR-210-3p directly targeted EFNA3 in HUVECs.

### Inhibition of miR-210-3p Abolished N-EV-mediated Cardiac Function Protection in a Rat MI Model

**Fig. 7:**
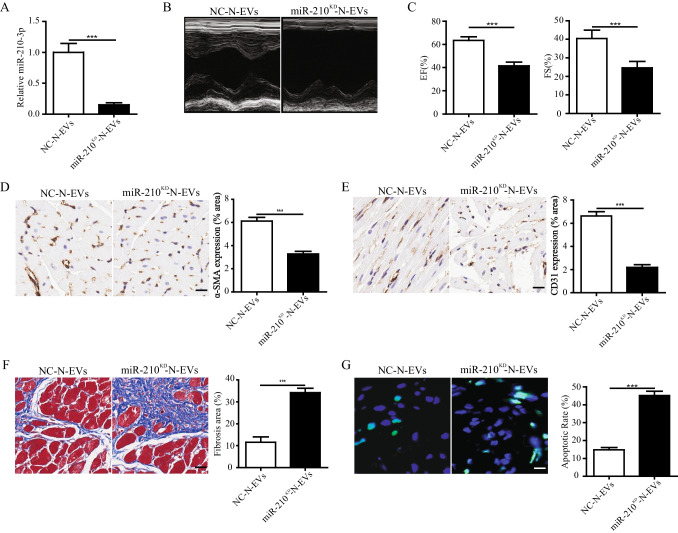
Inhibition of miR-210-3p impaired N-EV-mediated cardiac function protection in a rat MI model. **A** MiR-210-3p expression was detected by qRT-PCR in EVs isolated from the N-MSCs treated with 100 nM miR-210-3p inhibitor or negative control (NC). **B** Representative echocardiogram of rat heart in different groups at 28 days post-MI. scale bar = 25 μm. **C** Significantly enhanced LVEF, LVFS in rats transplanted with NC-N-EVs compared with miR-210^KD^-N-EVs groups. **D** α-SMA positively stained arterioles in the infarct area. scale bar = 25 μm. **E** CD31 positively stained capillaries at the border zone. scale bar = 25 μm. **F** Representative transverse heart sections analysed with masson trichrome staining. scale bar = 25 μm. **G** TUNEL staining at the border zonescale. scale bar = 25 μm. n = 5 for each group. Data were expressed as the mean ± SD. ^*^P < 0.05, ^**^P < 0.01, ^***^P < 0.001

To identify whether miR-210-3p was responsible for the beneficial effects of N-EVs, the miR-210-3p expression in N-EVs was knocked down (called miR-210^KD^-N-EVs) via miR-210-3p silencing (Fig. 7A). MiR-210^KD^-N-EVs or its control (NC-N-EVs) were identified and characterized by using transmission electron microscope (TEM), nanoparticle tracking analysis (NTA) and western blot (Fig. S3A-C). The above results suggested that there is no difference in characterization between miR-210^KD^-N-EVs and NC-N-EVs. Then, miR-210^KD^-N-EVs or NC-N-EVs were injected into rat MI model. After four weeks, our results showed that the percentage of LVEF and LVFS in miR-210^KD^-N-EVs group was significantly lower than in NC-N-EVs-treated mice (Fig. 7B, C). The expression of α-SMA and CD31 in miR-210^KD^-N-EVs group were clearly lower than in the NC-N-EVs group (Fig. 7D, E). Masson staining results indicated that the area of fibrosis in miR-210^KD^-N-EVs group significantly higher than NC-N-EVs group (Fig. 7F). Furthermore, the knocked down of miR-210-3p in N-EVs reduced its ability to repress cell apoptosis (Fig. 7G). These findings demonstrated that miR-210-3p knockdown impaired N-EV-mediated cardiac function protection in a rat MI model.

## Discussion

In the present study, we first reported that N-EVs more significantly promoted the proliferation, migration, and tube formation of HUVECs. Meanwhile, our results indicated N-EVs was more superior in improving cardiac function, reducing cardiac fibrosis and increasing angiogenesis compared with M-EVs. Further analysis showed that the cardioprotective effect of N-EVs was mediated by miR-210-3p targeting EFNA3. These findings uncover a previously unrecognized the role of EVs derived from NMN-pretreated hUCMSCs in cardioprotection in MI animal models and how this EVs mediator exerts cardioprotective effects.

Angiogenesis plays an important role in salvaging ischemic tissues and promoting cardiac function [28]. However, angiogenesis is insufficient after myocardial infarction. Therefore, the stimulation of angiogenesis is the main strategy in the treatment of patients with myocardial infarction [29]. MSCs and their EVs have been shown to have good cardioprotective effects by stimulating cardiac angiogenesis [30–33] but how to further enhance their potential therapeutic effects is equally important. Several recent studies have shown that MSCs after intervention are more protective of the heart. A recent study showed that macrophage migration inhibitory factor facilitates the therapeutic efficacy of mesenchymal stem cells derived EVs in acute myocardial infarction through upregulating miR-133a-3p [22]. Another study showed that hemin enhances the cardioprotective effects of mesenchymal stem cell-derived EVs against infarction via amelioration of cardiomyocyte senescence[34]. Xuan et al. also reported that extracellular vesicles from notch activated cardiac mesenchymal stem cells promote myocyte proliferation and neovasculogenesis[35]. Liu et al. reported that identification of a CTRP9 C-Terminal polypeptide capable of enhancing bone-derived mesenchymal stem cell cardioprotection through promoting angiogenic exosome production[36]. Recently, Ning et al. found that FNDC5 pre-conditioning bone marrow-derived mesenchymal stem cells (BMMSCs) could secrete more EVs, which protect myocardial infarction by anti-inflammation and macrophage polarization via NF-κB signaling pathway and Nrf2/HO-1 axis [37]. Similarly, our findings confirmed that EVs generated by hUCMSCs cultured under NMN preconditioned have better cardioprotective ability. N-EVs may represent a promising therapeutic option for the treatment of myocardial infarction. Although increasing evidences showed that modified or pretreated MSC-EVs have great potential for cardioprotection in vitro and in vivo. However, more extensive testing and validation of EVs therapeutics are needed to assure safety and efficacy[38].

Numerous studies have confirmed that MSC-EVs modulated the multiple biological activity of target cells by transferring specific miRNAs [39–41]. The results of this study supported a model in which EVs released by N-MSCs produced pro-angiogenic effects that protect cardiac function through a mechanism involving miR-210-3p. In this current study, expression profiles of miRNAs in N-EVs revealed that miR-210-3p was significantly increased in N-EVs compared with M-EVs, and quantitative PCR experiment results also revealed that the expression of miR-210-3p was significantly increased in HUVECs after N-EVs stimulated compared with M-EVs. However, further studies are needed to investigate whether the expression of other miRNAs is significantly increased in HUVECs. Growing evidence has shown that miR-210-3p has a very important role in promoting angiogenesis. MiR-210-3p promoted blood vessel formation through various mechanisms such as EFNA3, RGMA, SMAD4 and STAT6[42–44]. Consistent with our research, overexpression of miR-210-3p could promote the angiogenesis ability of HUVECs. We confirmed this through the luciferase report experiment. However, we needed further study to prove that EFNA3 were the direct targets of miR-210-3p to promote angiogenesis in the future.

The present study had many limitations. First, although intramyocardial injection could deliver nucleic acid-containing vesicles or drugs to the site of heart injury effectively, it could also cause additional damage to the heart, and the injected vesicles would also have the risk of leakage. Second, we had only seen significant improvements in cardiac function with intramyocardial injection of EVs in rats, however, we still needed to label EVs in order to observe the distribution of EVs in the heart after injection. In addition to endothelial cells that could ingest EVs, EVs might also be absorbed by other cells, leading to additional targeting effects. Genetic or chemical modification of EVs might be one of the ways to solve this problem. Third, our study only illustrated the effects of miR-210-3p in EVs on improving cardiac function after myocardial infarction. Moreover, miRNA sequencing results showed that EVs also had other miRNAs, and EVs also contained proteins, lipids, etc. The effects of these substances on heart function after myocardial infarction needed to be further studied. Fourth, multiple studies demonstrate angiogenesis the infarcted heart markedly improved subsequent the extent of fibrosis[45, 46] and as our results demonstrate, the degree of myocardial fibrosis significantly reduced after N-EVs treatment. Nevertheless it has been shown that MSC-EVs could directly have an anti-fibrotic effects in more organs. For example, Wang et al. [47]have demonstrated that Bone marrow mesenchymal stem cell-derived extracellular vesicles containing miR-181d protect rats against renal fibrosis by inhibiting KLF6 and the NF-κB signaling pathway. Another previous study also reported that Human bone marrow mesenchymal stem cell-derived extracellular vesicles inhibit shoulder stiffness via let-7a/Tgfbr1 axis[48]. Therefore, additional experimental validation is required to verify whether N-EVs could directly target to fibroblasts in the heart and reduce the fibrosis in the hearts of the rats after MI.

Taken together, our study demonstrated that EVs derived from hUCMSCs could improve cardiac function by promoting angiogenesis, promoting proliferation, inhibiting apoptosis, reducing fibrosis, and improving cardiac function, which is further enhanced after NMN treatment. MiR-210-3p and its downstream signal pathway were involved in the biological activities of N-EVs. These findings highlighted that N-EVs is a new therapeutic option for MI patients.

## Methods

### Preparation and Identification of hUCMSCs

The hUCMSCs were purchased from Haixing Biosciences (Suzhou, Jiangsu, China). The hUCMSCs were cultured in accordance with a previously described method[45]. Afterward, the adipogenic, osteogenic, chondrogenic capacity of these cells was performed. Passages 3–7 of hUCMSCs were cultured in osteogenic, adipogenic or chondrogenic differentiation medium (Cyagen, China, HUXUC-9004) as described by manufacturer. Oil red O staining, Alizarin red staining and Alcian blue staining were used to evaluate adipogenesis, osteogenesis, and chondrogenesis. Detection of hUCMSCs surface markers by a FACSVerse instrument (BD Bioscience, San Jose, CA, USA) is performed as follows. The hUCMSCs were stained with human anti-CD14, anti-CD19, anti-CD34, anti-CD45, anti-CD73, anti-CD90, anti-CD105, or anti-HLA-DR. Identical concentrations of PE-conjugated mouse IgG isotype antibodies were used as negative controls (all from BD Biosciences). Data were analyzed using the software FlowJo V10 (FlowJo).

### The Extraction and Identifcation of M-EVs and N-EVs

M-EVs and N-EVs were prepared by referring to the previous experimental methods [49, 50]. We used the dose as previously described[51], hUCMSCs were cultured in stem cell culture medium (Cyagen, Guangzhou, China) with or without 2.25 μM NMN for 48 h. Afterwards cells were washed 3 times with PBS, all hUCMSCs were cultured in serum free culture medium supplemented with 1% of PS for an additional 24 h. Subsequently, the M-EVs or N-EVs were isolated from the supernatant of hUCMSCs by ultracentrifugation (Beckman Coulter Optima L-100 XP ultracentrifuge, Miami, FL) as our previously described[23]. Briefly, the culture supernatants were centrifuged at 300 × g for 10 min to remove living cells and 2,000 × g for 20 min to remove cell debris and dead cells. Subsequently, medium was collected and filtered through a 0.45 μm filter. The collected supernatant was then ultracentrifuged at 100,000 × g for 1.5 h at 4 °C. The pellet was then washed with ice-cold PBS, followed by a second ultracentrifugation, and resuspended in PBS for further use.

For TEM analyses, the purified M-EVs and N-EVs was placed on non-glow-discharged carbon-coated grids for 10 min and then negatively stained for 1 min with 2% (w/v) uranyl acetate. Excess adherent negative stain was removed from grid by wicking with filter paper, and the dried grids were viewed using a transmission electron microscope (JEM-1200EX; JEOL Ltd, Tokyo, Japan). Dynamic light scattering was performed with M-EVs or N-EVs to generate size distribution data that was analyzed using Zetasizer Software.

### Analysis of EVs Uptake in HUVECs

CM-Dil (red, 1 μM) was added to M-EVs or N-EVs (10 μg/mL) for labeling, and the excess dye was removed using ultracentrifugation and washed twice. HUVECs were cultured in medium containing DIO (green) solution for 20 min at 37 °C and washed twice with PBS. DIO-labeled HUVECs were incubated with Dil-labeled EVs for 2 h and then fixed with 4% paraformaldehyde (PFA) at room temperature for 10 min, washed with PBS for three times. Subsequently, HUVECs incubated with DAPI for 5 min at room temperature. All reagents were from Invitrogen (Carlsbad, CA, USA). Samples were observed via Nikon Eclipse Ti confocal laser scanning microscope.

### Preparation of miR-210-3p^KD^-N-EVs and NC-N-EVs

The hUCMSCs were transfected with miR-210-3p inhibitor or negative control (100 nM; GenePharma, Shanghai, China) by using Lipofectamine 2000 (Invitrogen), as our previously described[23]. Afterwards, hUCMSCs were cultured with serum-free culture medium at 37 °C after NMN preconditioned. Subsequently, cell supernatants were collected and the EVs were isolated using the protocol described before. The EVs called miR-210-3p^KD^-N-EVs and NC-N-EVs, respectively.

### Cell Culture and Treatments

Human Umbilical Vein Endothelial Cells (HUVECs; FuDan IBS Cell Center, Shanghai, China) were cultured in DMEM supplemented with 10% FBS and 1% penicillin–streptomycin at 37 °C and 5% CO_2_. The culture medium was replaced every 2 days, and cells were subcultured or subjected to experimental procedures at 80–90% confluence. HUVECs were seeded in 6 or 96 wells plates and treated with PBS, M-EVs or N-EVs (1 × 10^3^ /cells) for 24 h. Then, the cells were subjected to culturing conditions for another 12 h. Subsequently, the cell viability assay, tube formation of HUVECs assay, wound healing assay were using to evaluated the differential effects of M-EVs and N-EVs on HUVECs.

### Cell Viability Assay

The cell proliferation and activity of HUVECs was determined using the CCK-8 assay. In brief, HUVECs (5 × 10^3^ cells/well) were seeded into 96-well culture plates and incubated overnight and then the cells treated with M-EVs or N-EVs for 24 h. Afterwards, HUVECs were cultured for another 12 h. After incubation, 10 μL CCK-8 was added, and the absorbance was measured at 450 nm by a spectrophotometer set (ELx800, BioTek, Winooski, VT, USA).

### In vitroTube Formation Assay

Angiogenesis of HUVECs was assessed using a capillary tube forming assay. Matrigel (BD Biosciences) was diluted 1:1 with DMEM and a total of 50 µL of the mixture was used to precoat each well of 96-well plates for 30 min. HUVECs (3 × 10^4^ cells/well) were seeded in triplicate onto matrigel plates and then cells were incubated at 37 °C for 3 h. Capillary-like structures were evident and counted using inverted phase contrast microscope and the degree of tube formation was analyzed by counting the number of complete tubes.

### Wound Healing Assay

HUVECs were incubated in 6-well plates in complete culture medium and cultured overnight to reach full confluence as a monolayer. Wounds were created using a 10-µL sterile pipette tips and the plates were then washed twice with PBS to removed the detached cells. The cells were photographed immediately (time 0) and 24 h after scratching using a phase-contrast microscope. The width of the wound was measured using ImageJ software (NIH, USA) and reported as percentage final wound width/initial wound width. The experiment was replicated three times.

### Western Blotting

HUVECs were lysed in cold 1 × Cell Lysis Buffer (Cell Signaling Technology, USA) supplemented with 1 mM PMSF (Sigma) at 4 °C for 30 min. The M-EVs and N-EVs were lysed in 20 μL lysis buffer at 4 °C for 10 min. Total protein concentration was determined using the BCA protein assay kit (Pierce, USA). Western blotting was performed according to the standard protocol, as our previously described [38]. The antibodies used were as follows: CD63 (1:1000, ab134045, Abcam), TSG101 (1:1000, ab133586, Abcam), glyceraldehyde-3-phosphate dehydrogenase (GAPDH, 1:1000, 5174, Cell Signaling Technology), EFNA (1:1000, 33,810, Signalway Antibody). The secondary antibody was horseradish peroxidase-conjugated antibody. Protein bands were visualized by chemiluminescence using enhanced chemiluminescence substrate (Millipore Corporation, Bedford, MA) and the blots were analyzed using Image J software (NIH, USA).

### RNA Sequencing and Analysis

M-EVs and N-EVs were prepared by ultra-high-speed centrifugation and RNAs were extracted using a Norgen Exosome RNA Isolation Kit (Norgen) in accordance with the manufacturer’s instructions. The Quality of RNA was evaluated using the Agilent 2100 bioanalyzer (Agilent, CA, USA) with the RNA 6000 Nano LabChip kit. RNA (10 ng) was used to prepare a small RNA library according to the protocol of TruSeq Small RNA Sample Prep kits (Illumina, San Diego, CA, USA). Single-end 50-bp sequencing was performed using Illumina Hiseq2500 at the LC-BIO (Hangzhou, China) following the manufacturer’s recommended protocol. We utilized the bioinformatics database Targetscan 7.2 to predict the genes targeted by the differentially expressed miRNAs.

### Cell Transfection with miRNA mimics/inhibitor

MiR-210-3p mimics/mimics-negative control (NC) and their inhibitors/inhibitor-NC were synthesised and purified by GenePharma. Transfections were as follows: the HUVECs were plated onto 6 well-plates until 70–80% confluence. Then, HUVECs were transfected with miR-210-3p mimics/mimics-negative control (NC) or their inhibitors/inhibitor-NC (GenePharma, Shanghai, China) at a concentration of 100 nM using Lipofectamine 2000 (Invitrogen) with Opti-MEM media (Invitrogen). The cell culture medium was changed 6 h after transfection. All transfections were carried out according to manufacturers’ instructions.

### Real-time Quantitative PCR (RT-qPCR) Analysis

Total RNAs from HUVECs were isolated using the Trizol reagent (Invitrogen) and reverse transcribed into complementary DNA using the HiScript III RT SuperMix for qPCR (R302-01, Vazyme, Nanjing, China) following the manufacturer’s description. MiRNAs was extracted from the M-EVs and N-EVs using a mirVana RNA isolation kit (Ambion, Austin, TX), synthesis of cDNA and qRT-PCR were performed using All-in-One miRNA qRT-PCR Detection Kit (GeneCopoeia, Rockville, MD, USA) following the manufacturer’s protocol. Then, quantitative PCR was performed using ChamQ SYBR qPCR Master Mix (Low ROX Premixed) (Q331-02, Vazyme, Nanjing, China) for mRNA and 2 × All-in-One qPCR Mix (GeneCopoeia) for miRNA based on LightCycler96 PCR system (Roche, Inc., Switzerland). The primers for real-time PCR (miR-210-3p, miR-210-3p, miR-708-3p, miR-301a-5p, miR-491-5p, miR-301b-3p, miR-4485-3p, miR-548, miR-1-3p, miR-2355-3p and U6) all were purchased from GeneCopoeia. The relative expression of mRNA and miRNA was evaluated by the 2^−ΔΔCt^ method. The mRNA quantification of qPCR was normalized to GAPDH expression and miRNA quantification was normalized to U6.

### Luciferase Activity Assay

HUVECs were plated in 24-well plates overnight and then were co-transfected with 500 ng pmiR-RB-report-h-EFNA3-3′-UTR (wild type and mutant type) and 100 nM miR-210-3p mimic, 100 nM miR-210-3p inhibitor and their negative controls (GenePharma) using Lipofectamine 2000 (Invitrogen), followed by culturing with conditioned medium[45]. Cells were lysed 48 h after transfection and luciferase activity was then determined by the Luc-Pair™ Duo-Luciferase Assay Kit 2.0 (GenePharma). Luciferase activity was normalized by Renilla/Firefly luciferase signal in HUVECs.

### TdT-mediated dUTP Nick End Labeling (TUNEL) Staining

A terminal deoxynucleotidyl transferase dUTP nick end labeling (TUNEL) assay kit (Roche, Basel, Switzerland) was used for tissue apoptosis following the manufacturer’s instructions. Cell nuclei were stained with DAPI. The cells with TUNEL-positive nuclei were considered apoptotic. Samples were analyzed using a confocal laser scanning microscope (Zeiss LSM510 Meta Confocal Microscope, Germany). Finally, apoptotic index was calculated as a percentage of apoptotic nuclei to total nuclei. Each experiment was performed in technical triplicate.

### Rat MI Model and Assessment of Heart Functions

The investigation conforms NIH Guide for the Care and Use of Laboratory Animals. The experimental procedures followed the guidelines of the Institutional Animal Care and Use Committee of Nanjing Medical University and were approved by the Nanjing Medical University Animal Care and Use Committee Sprague Dawley (SD) rats (male, 6–8 weeks) were purchased from Weitong Lihua Experimental Animal Center (China). All rats were anaesthetized with 1% sodium pentobarbital (50 mg/kg) via intraperitoneal injection. A left thoracotomy was performed between the 3rd and 4th intercostal space under sterile conditions. The left anterior descending coronary artery was identified and ligated 1.5 mm below the left atrium auricula. Successful MI was verified via epicardial blanching, and then 50 uL M-EVs or N-EVs (2 × 10^9^ particles [52]) were divided into 4 equal portions and injected in the border zone of infarction area 30 min after ligation[47]. Post-operative analgesia was given as required (Carprofen, 10 mg/kg) to ensure that they did not experience any type pain. All surgeries and subsequent analyses were conducted in a blinded manner.

Four weeks after surgery, transthoracic echocardiography (Vevo 2000 high-resolution micro-imaging system) was performed to evaluate cardiac function. Left ventricle (LV) function was measured by M-mode echocardiography in the short-axis view at the mid-ventricular level. Left ventricular ejection fraction (LVEF) and left ventricular fractional shortening (LVFS) were measured using the Vevo 2000 workstation software to evaluate heart function.

### Masson’s Trichrome Staining and Immunofuorescence

The rats were euthanized at week 4 and their hearts were harvested for histological evaluations. Hearts were prepared for paraffin tissue sectioning after fixation with 4% PFA. All embedded tissues were sectioned for 5 μm thick and were stained with masson’s trichrome. Images were captured by scanning electron microscope (SU8010, Japan) and the collagen area of the infarct zone (CAIZ) is expressed as the ratio of the area of blue staining in the infarct zone to staining in the noninfarct zone using Image J software. For immunofuorescence analyses, heart sections were stained with a primary antibody, rat monoclonal CD31 (ab182981, Abcam, Cambridge, UK) and α-smooth muscle actin (α-SMA, ab124964, Abcam, Cambridge, UK), and the secondary antibody (ab6721, Abcam, Cambridge, UK).

### Statistical Analysis

All statistical analysis were performed with GraphPad Prism (Version 7.0; La Jolla, CA). All results are shown as the mean ± standard deviation (SD). The Student t test or one-way analysis of variance (ANOVA) was employed for comparisons between groups. P values < 0.05 were considered statistically significant (*P* < 0.05).

## Supplementary Information

Below is the link to the electronic supplementary material.

Supplementary file1 (DOCX 839 KB)

## Data Availability

The raw data supporting the conclusions of this article will be made available by the corresponding authors, without undue reservation, to any qualified researcher.
