# Supplementary material for: Extracellular Vesicles from NMN Preconditioned Mesenchymal Stem Cells Ameliorated Myocardial Infarction via miR-210-3p Promoted Angiogenesis
Source: Stem Cell Rev Rep. 2023 Jan 25;19(4):1051–66. doi: 10.1007/s12015-022-10499-6 (PMC10185590; doi:10.1007/s12015-022-10499-6)
Supplement: Supplementary file 1 — Supplementary file1 (DOCX 839 KB) [file 12015_2022_10499_MOESM1_ESM.docx]

**Supplemental results**

**Figure S1. The results of CCK8 assays to assess the proliferation of MSCs**

**Figure S2. Identification of NMN preconditioned hUCMSCs**


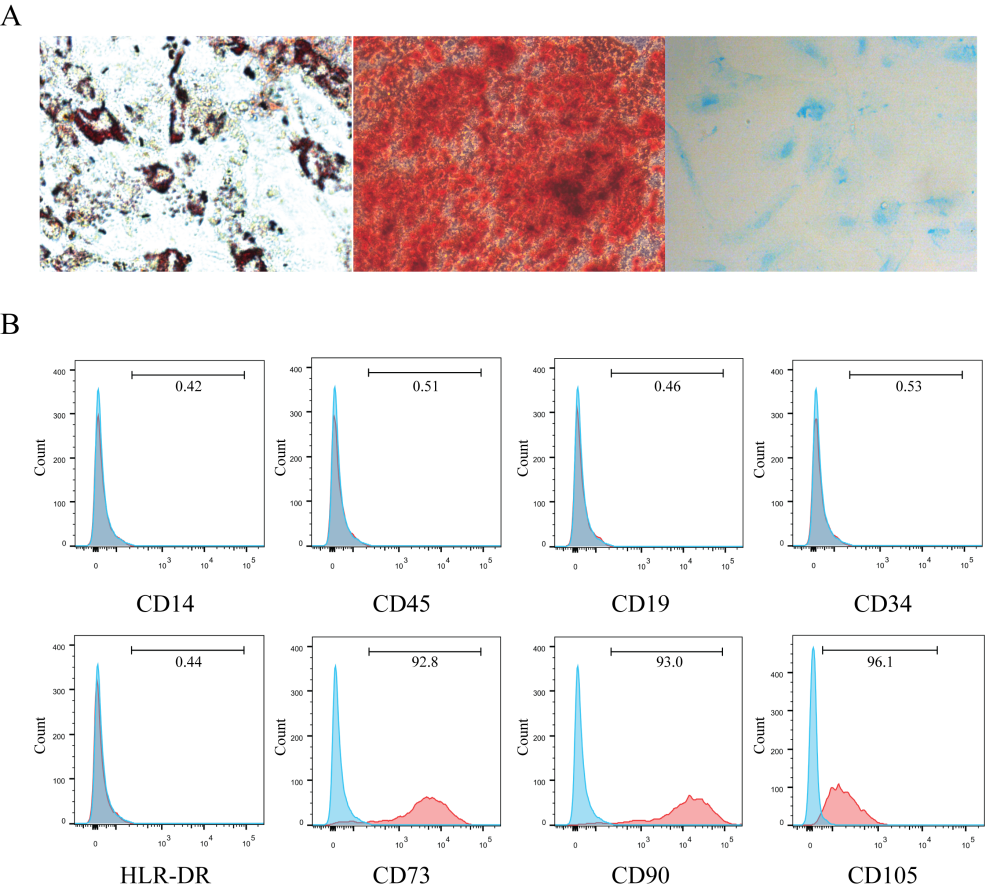


A. Representative photographs of osteocyte, adipocyte and chondrocyte differentiation of hUCMSCs cultured in the differentiation media.

B. Flow cytometric analysis of cell surface markers of hUCMSCs (CD14, CD45, CD19, CD34, HLA-DR, CD73, CD90, and CD105).

**Figure S3. Characterization of NC-N-EVs and miR-210^KD^-N-EVs**


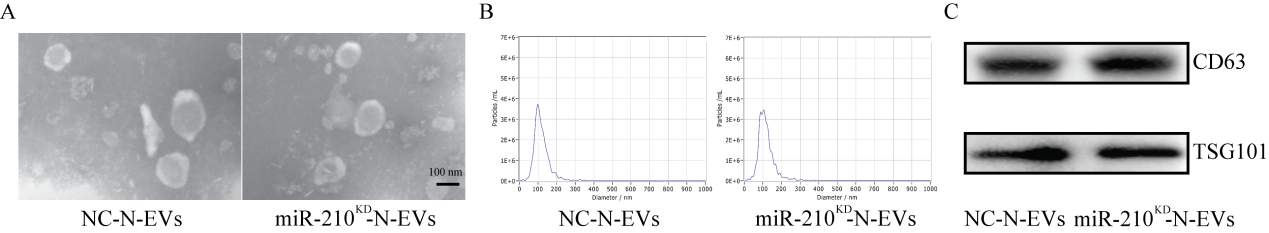


A. Typical images of TEM of NC-N-EVs and miR-210^KD^-N-EVs.

B. The particle size distribution and concentration of NC-N-EVs and miR-210^KD^-N-EVs by NTA.

C. Western blot analysis of TSG101 and CD63 expression in NC-N-EVs and miR-210^KD^-N-EVs.
